# Growth but Not Corticosterone, Oxidative Stress, or Telomere Length Is Negatively Affected by Microplastic Exposure in a Filter‐Feeding Amphibian

**DOI:** 10.1002/jez.70005

**Published:** 2025-06-22

**Authors:** Colette Martin, Katharina Ruthsatz, Ivan Gomez‐Mestre, Pablo Burraco

**Affiliations:** ^1^ Zoological Institute Technische Universität Braunschweig Braunschweig Germany; ^2^ Institute of Biodiversity, Animal Health and Comparative Medicine University of Glasgow Glasgow UK; ^3^ Ecology, Evolution, and Development Group, Department Ecology and Evolution Doñana Biological Station, CSIC Seville Spain

**Keywords:** ageing, amphibian decline, glucocorticoids, plastic pollution, redox status, *Xenopus laevis*

## Abstract

Microplastics (MPs) are of increasing global concern for species inhabiting aquatic habitats. However, the mechanisms behind animal responses to MPs still require comprehensive exploration. Amphibians are the most threatened vertebrate group with most species having a complex life cycle, commonly with an aquatic larval stage. Here, we investigated whether exposure to an environmentally relevant concentration of MPs affects the growth of filter‐feeding larvae of the African clawed frog (*Xenopus laevis*), and the consequences for their stress physiology (corticosterone [CORT] levels), or health and ageing physiology (oxidative stress and telomere length, the latter in the liver and gut). We conducted a 3 × 2 experiment with three levels of fiber exposure (fibers absent ‐control‐, and MP and cellulose fiber treatments), and two stress levels (CORT absent –control‐, and CORT present simulating a stressful condition). We observed a negative impact of MP exposure on larval growth; however, this did not alter the CORT levels, oxidative stress. or telomere length. Our study shows that realistic concentrations of MPs are not enough to induce major alterations on the stress or health and ageing physiology of a filter‐feeding amphibian. Whether compensatory growth responses during the post‐metamorphic stages could lead to detrimental effects later in life should be explored in amphibians and other organisms with complex life cycles.

## Summary

- Environmentally realistic concentrations of microplastic fibers reduced growth in amphibian larvae, but did not alter physiological stress or ageing biomarkers.
- Exposure to both microplastics and stress hormone (corticosterone) did not produce additive or synergistic negative effects.
- Short‐term microplastic exposure may not impair health‐related physiology in early amphibian stages, but potential long‐term consequences deserve further investigation.

## 1 Introduction

Human‐driven activities expose wildlife to a myriad of stressors, including altered temperatures, habitat loss, and pollution (Lee et al. 2023). These conditions have the potential to impair vital processes such as metabolism, growth, development, or reproduction, which finally can compromise organismal health and survival (Román‐Palacios and Wiens 2020; Sheridan and Bickford 2011). Consequently, anthropogenic disturbances may adversely affect fitness and lead to population declines, thereby contributing to the current global biodiversity loss (Díaz et al. 2019). Some physiological pathways have been recently presented as markers of individual fitness and health or ageing (e.g., Angelier and Wingfield 2013; Burraco et al. 2020; Lemaître et al. 2022; Schoenle et al. 2021), and thus they can improve our understanding of eco‐toxicological processes and advance conservation management plans.

A central physiological mechanism mediating animal responses to habitat changes is the hypothalamic–pituitary–adrenal/interrenal axis (Crespi et al. 2013; Sapolsky et al. 2009; 2000). When internal homeostasis is disrupted, the activation of this neuroendocrine stress axis ultimately results in the secretion of glucocorticoids (GCs). Cortisol is the predominant GC in most primates and teleost fish, and corticosterone (CORT) in most amphibians and sauropsids (Angelier and Chastel 2009; Glennemeier and Denver 2002; Schreck and Tort 2016; Suarez‐Bregua et al. 2018). The release of GC hormones mobilizes energy stores to meet increased metabolic demands to regulate metabolism and nutrient homeostasis (Kirschman et al. 2017; Sapolsky et al. 2000). In addition to energy‐related functions, GCs play a pivotal role in developmental processes across many taxa (rev. in Crespi et al. 2013). For instance, in animals with at least two discrete life stages, GCs can plastically accelerate development and growth, allowing the transition to the next life stage to escape suboptimal habitats (Crespi and Warne 2013; Denver 2009; Gomez‐Mestre et al. 2013). GC release and subsequent enhanced development can include cascading effects at different molecular levels such as the induction of an oxidative state (Costantini 2014, 2019), which has the potential to damage the structure and function of molecules within the cell, including DNA (Halliwell 2007; Monaghan et al. 2009). Particularly, oxidative stress is thought to lead to telomere shortening (Armstrong and Boonekamp 2023; Chatelain et al. 2020). When telomeres become critically short, cell apoptosis is induced (Lin and Epel 2022). Therefore, telomere shortening is considered a hallmark of cellular and organismal ageing (López‐Otín et al. 2023) and can predict fitness and survival odds (Eastwood et al. 2023; Q. Wang et al. 2018; Wilbourn et al. 2018). Integrating these health and ageing‐related parameters into ecotoxicology and evolution will therefore enable us to better understand the molecular processes governing human‐driven life history shifts.

Environmental pollution resulting from, for instance, industrial waste, agricultural chemicals, or urban runoff, is dramatically pushing many natural populations to the brink of extinction (Noyes and Lema 2015; Sigmund et al. 2023; Wake and Vredenburg 2008). Aquatic habitats are particularly threatened by pollutants since these environments act as a sink for almost all kind of contaminants (Kumar et al. 2021; Mushtaq et al. 2020; Rzymski et al. 2017). Among several other pollutants, the presence of microplastics (MPs) is an increasing global concern for aquatic organisms due to large production volumes, continuous and worldwide release, and long‐term environmental persistence in ecosystems (L. Li et al. 2018; PlasticsEurope 2022; Windsor et al. 2019). MPs are defined as synthetic polymer particles with diameters ranging from 1 μm to 5 mm (Hartmann et al. 2019; Thompson et al. 2004). The source of MPs is diverse as they derive from either primary plastic used for purposes such as personal care products, production pellets, or textiles, and from secondary plastics such as debris of plastic items, fishing nets, or tires (Horton et al. 2017). Despite being most abundant in the marine environment (rev. in Y. Wang et al. 2022), freshwater environments have been identified as a key pathway in the transport of MPs from terrestrial to marine ecosystems (rev. in Stanton et al. 2020). Given their size range, MPs fall within the prey range for a variety of aquatic animals and thus they can be mistakenly ingested (Franzellitti et al. 2019) possibly resulting in adverse organismal effects, including reductions in growth, reproduction, and survival (rev. in Li et al. 2023; Prokić et al. 2019). However, the mechanisms driving these major effects of MPs are not very well understood in many taxa.

Amphibian larvae represent an ideal study system to investigate the mechanistic consequences of MP pollution on aquatic organisms due to their nonselective feeding mode, hormone‐regulated development, semi‐permeable skin, and limited dispersal capacity (Gonçalves et al. 2024; Ruthsatz and Glos 2023; Shi 2000). Amphibians coping with stressors activate the HPI axis which leads to CORT and thyroid hormones release (Kirschman et al. 2017; Kulkarni and Buchholz 2014). At the larval stage, higher CORT levels are known to accelerate development and growth rates, then allowing the transition to the terrestrial post‐metamorphic stage to avoid suboptimal habitat conditions (Crespi and Denver 2005; Crespi and Warne 2013; Denver 2009). Amphibian larvae of many species are known to express developmental plasticity in response to conditions such as pond drying (e.g., Kulkarni et al. 2017; O'regan et al. 2014; Székely et al. 2017), increased water temperature (Sinai et al. 2022) or pathogen presence (Warne et al. 2011), all responses resulting in higher CORT levels. Also, altered developmental trajectories often come with trade‐offs, as faster development can lead to reductions in body condition, locomotor performance, or immunocompetence (Burraco, Rendón, et al. 2022; Gervasi and Foufopoulos 2008; Ruthsatz, Dausmann, et al. 2019; Ruthsatz et al. 2020), as well as shifts in fitness‐related traits such as the timing of sexual maturation (Burraco, Torres‐Montoro, et al. 2023). These developmental constraints may contribute to the fact that approximately 41% of all amphibian species are threatened with extinction, with climate change, habitat loss, and environmental pollution acting as major drivers of those declines (Luedtke et al. 2023; Stuart et al. 2004). Furthermore, the baseline levels of endogenous CORT in amphibians can be easily manipulated by administering exogenous CORT in water, allowing it to be taken up via skin absorption (Glennemeier and Denver 2002). Such use of exogenous CORT has allowed to infer causality of this hormone in developmental and growth processes during pre‐metamorphic life stages (Denver 2009; F. Hu et al. 2008; Kulkarni and Gramapurohit 2017).

In relation to MP contamination, research has shown that polymer ingestion has detrimental effects on amphibian larval growth (Balestrieri et al. 2022; but not: De Felice et al. 2018), behavior (da Costa Araújo and Malafaia 2020; but not: Scribano et al. 2023), body condition (Boyero et al. 2020), developmental rate (Ruthsatz et al. 2022; Ruthsatz, Rico‐Millan et al. 2023), and metabolism (Ruthsatz, Schwarz, et al. 2023). Furthermore, MP accumulation has been found in the digestive tract of amphibians following ingestion (L. Hu et al. 2016), which may result in carry‐over effects, particularly in filter‐feeding species potentially ingesting large amounts of MPs present in the water body. However, only three recent studies have addressed the impact of MPs on some physiological markers of health and stress in amphibians (da Costa Araújo et al. 2023; Attademo et al. 2023; Ruthsatz, Schwarz, et al. 2023), and thus further comprehensive approaches will finally determine the actual impact of MPs on the condition of amphibians and other aquatic organisms.

Here, we investigated whether MP ingestion has consequences for physiological parameters in larvae of the filter‐feeding African clawed frog (*Xenopus laevis*). The experimental set‐up consisted of a 3 × 2 design, including three levels of fiber exposure (a no‐fiber control, and MP and cellulose fiber treatments), and two levels of CORT level manipulation (presence/absence of exogenous CORT). We predicted that MP ingestion would decrease larval growth and that it would result in impaired physiology, including increased CORT levels and oxidative stress (or, alternatively, buffered oxidative stress through increased antioxidant responses), and telomere shortening.

## 2 Materials and Methods

### 2.1 Experimental Design and Animal Husbandry

Two egg clutches of *X. laevis* were obtained from the Andalusian Center for Development Biology (CABD, Pablo de Olavide University and CSIC), and kept at 24°C in a single bucket filled with de‐chlorinated water. When larvae reached the developmental stage NF40 (free‐swimming stage; Faber and Nieuwkoop 2020), we randomly allocated one individual to each of 102 round containers filled with 2.6 L of de‐chlorinated water (i.e., 17 tadpoles per treatment group). We pooled the tadpoles from the two clutches into a 10‐L bucket and randomly distributed them across the treatment buckets to minimize the chance of maternal or genetic influences on a given treatment. The experiment was conducted in a climate chamber set to 24°C and with a 14:10 h light:dark photoperiod. Larvae were fed a protein‐rich powdered fish food consisting of 50:50 sera micron powder and spirulina (Sera, Germany). This food is confirmed to be free of MPs and falls within the size range associated with MPs and cellulose (Ruthsatz, Schwarz, et al. 2023). Food was provided ad libitum, and sufficient rations were provided twice daily to ensure a constant and abundant food supply throughout the experiment. Larvae were maintained under these control conditions until they reached NF46, the point at which we started exposing larvae to the different treatments. Developmental stage NF46 was chosen as the starting point for the treatment exposure because, at this stage, the compact larval intestine forms, and the tadpoles begin filter‐feeding (Chalmers and Slack 1998; Chalmers and Slack 2000; Schreiber et al. 2005).

### 2.2 Fiber and CORT Exposure

We used polyethylene microplastic (MP) 34–50 μm particles (Sigma‐Aldrich; polyethylene powder, CAS number 9002‐88‐4). Polyethylene is one of the most commonly used polymers for creating plastic materials, also known to be a significant source of MPs in the wild (Horton et al. 2017; Karaoğlu and Gül 2020). Amphibians, particularly tadpoles, are exposed to polyethylene MPs worldwide (F. Hu et al. 2008; Karaoğlu and Gül 2020). Previous research has investigated the effect of polyethylene on amphibian life‐history and health (da Costa Araújo and Malafaia 2020; Ruthsatz et al. 2022; Ruthsatz, Schwarz, et al. 2023). We chose an MP concentration of 60 mg/L, following the procedure adapted from da Costa Araújo et al. (2020a). This concentration, as reported by Ruthsatz, Schwarz, et al. (2023), corresponds to a particle density of 1.0356–1.0675 × 10^7^ particles per litre. This density falls within the environmentally relevant range of surface water contamination with MP (Koelmans et al. 2019), and serves as an indicator of high pollution levels (da Costa Araújo et al. 2020a; da Costa Araújo et al. 2020b). We used cellulose (Sigma‐Aldrich; cellulose powder, CAS number 9004‐34‐6, mean particle size: 51 μm) at a concentration of 60 mg/L as a natural fiber control treatment in this experiment, that is, to control for a possible effect of exposure to fibers within the same size range ( ~ 50 μm in our case), but not of a plastic nature (Buss et al. 2021). Cellulose is a natural, biodegradable polymer derived from plant sources, and is micro‐plastic free (Buss et al. 2021; Ruthsatz, Schwarz, et al. 2023).

When tadpoles reached developmental stage NF46, the MP and cellulose fibers were added directly to the water in the experimental containers (i.e., 60 mg/L x 2.6 L water = 156 mg fibers per container). Air stone bubble diffusers ensured both constant and effective water aeration and the continuous dispersion of fibers within the water, preventing particle settling and the formation of an MP film on the water surface (Ruthsatz et al. 2022; Ruthsatz, Schwarz, et al. 2023). Five days after the start of fiber exposure, we dissolved CORT in absolute ethanol and added the stock solution to half of the buckets (*N* = 51) at a concentration of 100 nM (Burraco et al. 2015). Exogenous CORT added to the water is taken up directly by tadpoles via skin absorption (e.g., Glennemeier and Denver 2002). The water was fully renewed every other day to ensure the desired CORT concentration. During each water change, we used a separate net and bucket for each treatment, we wore cotton material clothing (i.e., polymer‐free) and bright blue nitrile gloves, and hair was tied back, to prevent cross‐contamination. An air purifying system (Philips AC2889/10, CADR 333 m^3^ × h^−1^) was used at all times in the experimental climate chamber to filter possible air contamination.

### 2.3 Sampling

Larvae were euthanised when they reached developmental stage NF57 (i.e., all five toes separated; Nieuwkoop 2020) via immersion in a lethal solution of tricaine methanesulfonate (2 g/L MS‐222, Ethyl 3‐amino‐benzoate methanesulfonate; Sigma‐Aldrich), buffered with 200 mg/L sodium bicarbonate (Cecala et al. 2007). We blotted dry and weighed each individual to the nearest 0.0001 g using a high precision balance (Ohaus VP‐114CN Voyager Analytical Balance, Spain). We measured snout‐vent length (SVL) and total length (TL) to the nearest 0.5 mm with a calliper. Then, for telomere length analyses, we dissected the liver and gut, blotted dry, and weighed (only the gut) separately to the nearest 0.0001 g using a high precision balance (Ohaus VP‐114CN Voyager Analytical Balance, Spain). The collected livers and guts were placed separately in 1.5 mL Eppendorf tubes, snap frozen, and stored at −80°C until used for telomere length quantification. The body remnants and tails were stored in 1.5 mL Eppendorf tubes and stored at −80°C for subsequent oxidative stress and CORT analyses.

### 2.4 Determination of Physiological Parameters

#### 2.4.1 Corticosterone (CORT) Assay

Hormone extraction took place in August 2023. We thawed and weighed each tail sample to the nearest 0.0001 g followed by homogenization in 13 × 100 mm glass tubes with 500 µL PBS buffer (AppliChem Panreac, Germany) using a homogenizer at ~17,000 rpm (Miccra D‐1, Germany). To collect the sample residue, we washed the tissue blender into the tube with additional 500 µL PBS buffer. Then, the blender was cleaned with ddH20, and 96% EtOH to avoid cross‐contamination. After homogenization, we added 4 mL of a 30:70 petroleum ether:diethyl ether dissolvent mixture (both from Sigma‐Aldrich, Germany) to each sample. Samples were vortexed for 60 s and subsequently centrifuged at 1800 g and 4°C for 15 min. Then, we snap‐froze samples in a dry ice ethanol bath for 5 min, collected the resulting top organic layer containing CORT, and placed each sample in a new 13 × 100 mm glass tube. We repeated all steps after homogenization to ensure maximum CORT extraction, and we pooled each recovered ether fraction into a single tube. We evaporated each sample with the help of a sample concentrator (Techne FSC400D; Barloworld Scientific, United Kingdom) consisting of a constant but gentle nitrogen flow. Finally, we resuspended lipids in 350 μL EIA buffer (Assay buffer, DetectX Corticosterone ELISA kit, K014‐H5, Arbor Assays, Ann Arbor, MI, USA) with the help of a vortex, then tubes were sealed with parafilm and incubated overnight in a fridge at 4°C.

We measured CORT levels using DetectX Corticosterone ELISA (Enzyme Immunoassay) kits from Arbor Assays (K014‐H5, Ann Arbor, MI, USA). ELISA assays have been validated and successfully used for CORT detection in several amphibians (e.g., *Lithobates sylvaticus*, Gavel et al. 2019; *Rana arvalis*, Mausbach et al. 2022; *R. temporaria*, Burraco et al. 2017; Ruthsatz et al. 2023a; *X. laevis*, Ruthsatz, Schwarz, et al. 2023). We used the 100 µL assay format for standard preparations and assays. CORT concentration was measured in triplicates for all samples on 96‐well plates according to the kit's instructions. The plates were read with a multimode microplate reader (MB‐580 3, Heales) at 450 nm. In total, we ran four plates. MyAssays online tools were used to calculate the hormonal concentration of samples based on calibration standards provided with the DetectX kit (https://www.myassays.com/arbor-assays-corticosterone-enzyme-immunoassay-kit-improved-sensitivity.assay). A new standard curve for calculation of the results was run for each plate. The mean coefficient of variation of triplicates for all samples was 19.91%. Intraplate variation was overall 24.6% and interplate variation was on average 30.01%. We kept all the measurements of triplicates with a coefficient of variation lower than or equal to 30.0% or with an absolute difference between mean and median lower than 2.5 pg (Ruthsatz, Rico‐Millan et al. 2023). Each plate also included a negative control with no CORT nor antibody. The average background CORT recorded in our negative control samples was subtracted from hormone samples. Average *R* ^2^ for the 4PLC fitting curve was 0.994.

#### 2.4.2 Oxidative Stress

We quantified the activity of four antioxidant enzymes (catalase, glutathione reductase, glutathione peroxidase, and superoxide dismutase), lipid peroxidation (malondialdehyde levels), and antioxidant status (reduced‐to‐oxidized glutathione ratio). The remnants of larvae (i.e., after collecting the tail for CORT quantification, and the gut and liver for telomere length measurements) were immersed in a buffered solution that inhibits proteolysis (Tris HCl 100 nM pH 7.8, EDTA 0.1 mM and 0.1% Triton X‐100) using a 1:4 (weight:volume) proportion, then were homogenized at 35,000 rpm with a Miccra homogenizer (Miccra D‐1). We centrifuged the homogenates at 20,800 g for 30 min at 4°C, aliquoted the supernatants into four different tubes, and stored them at −80°C until assayed ( < 4 months). To get levels of antioxidant enzymatic activities in relation to protein content in each sample, we determined total protein content using the autoanalyzer Cobas Integra 400 (Roche Diagnostics) with reagents purchased from RANDOX laboratory (Antrim, UK). We quantified the activity of glutathione reductase (in mU/mg protein), glutathione peroxidase (in mU/mg protein), superoxide dismutase (U/mg protein), and reduced‐to‐oxidized glutathione ratio using the autoanalyzer Cobas Integra 400 (Roche Diagnostics) with reagents purchased from RANDOX laboratory (Antrim, UK). Catalase activity (in U/mg protein) and malondialdehyde concentration (nmol/mL) were quantified according to standard colorimetric procedures (Cohen and Somerson 1969; Galván et al. 2010; see also: Burraco, Rendón, et al. 2022). Each sample was run in duplicate and intra‐sample CV% were 3.44, 6.27, 8.77, 0.47, and 3.75 for catalase, glutathione reductase, glutathione peroxidase, superoxide dismutase, and malondialdehyde, respectively.

#### 2.4.3 Relative Telomere Length

We extracted liver and gut genomic DNA using the PureLink DNA extraction kit (Invitrogen, ThermoFisher) following the protocol provided by the manufacturer. We quantified the concentration and quality of DNA using a Nanodrop spectrophotometer. DNA was stored at −80°C until assayed (< 6 months). Since changes in cell populations could drive telomere dynamics (Burraco, Hernandez‐Gonzalez, et al. 2023), in this study, we sampled the liver and gut for telomere length measurements because these tissues do not experience major developmental transformations between the developmental stages covered by the experiment (Nieuwkoop 2020).

Like in almost all vertebrates, *X. laevis* telomere sequences consist of TTAGGG tandem repeats. To quantify variation in telomere length, we followed a procedure used in many taxa that provides relative telomere length measurements, based on the qPCR threshold cycle value of telomere repeats and a control single‐copy gene. The genome of *X. laevis* presents almost no interstitial telomeric sequences (Meyne et al. 1990; Nanda et al. 2008), making the species a good candidate to estimate variation in telomere length through qPCR. We used primers designed for vertebrates to amplify their telomeric sequences: tel1b 5’‐ CGGTTTGTTTGGGTTTGGGTTTGGGTTTGGGTTTGGGTT‐3’ and Tel2b 5’‐ GGCTTGCCTTACCCTTACCCTTACCCTTACCCTTACCCT‐3’. We amplified a non‐variable copy reference gene (RAG‐1) as a control gene, using primers designed for *X. laevis* (Burraco, Hernandez‐Gonzalez et al. 2023): XENRAG1‐forward 5’‐GCTCCATCGTCAGAGTTTAG‐3’ and XENRAG1‐reverse 5’‐TGCTTCTGGTGGAGAATTAC‐3’. For each gene, amplifications were conducted in a LightCycler 480 Real‐Time PCR System (Roche) using a total of two 386‐well plates including samples randomly distributed. Conditions for telomere qPCRs were 15 min at 95°C for 15 s followed by 27 cycles of 95°C for 15 s, 58°C for 30 s and 72°C for 30 s, and a dissociation melting curve. Conditions for RAG qPCRs were 15 min at 95°C followed by 40 cycles of 95°C for 15 s and 60°C for 1 min, and a dissociation melting curve. Both primer combinations were used at 500 nM. Each qPCR plate contained a standard curve with five points serially diluted 1:1, a *no‐template control* containing all reagents except DNA, and samples run in triplicate. Within each triplicate, we discarded samples with a cycle threshold values higher than 0.5. Following MIQE guidelines (Bustin et al. 2009). We also discarded a possible influence of DNA concentration or quality on relative telomere length measurements by running linear correlations between Nanodrop concentration (*R* ^2^ = 0.001), 260/280 ratio (*R* ^2^ = 0.009), and 260/230 ratio (*R* ^2^ = 0.069). Intra‐plate CV% was 0.71 and 0.43 for telomere and RAG amplifications, respectively.

### 2.5 Statistical Analyses

All statistical analyses were conducted in R (R Development Core Team, version 4.21). We checked for data normality and homoscedasticity through Kolmogorov‐Smirnov (*lillie.test* function, *nortest* package) and Breusch‐Pagan tests (*bptest* function, *lmtest* package), respectively. To check for an effect of the different treatments on larval size, we conducted two linear models, one including body mass, and another including SVL as dependent variables. In both models, we included fiber and stress factors and their interaction as independent variables. We also explored the impact of fiber and stress exposure on gut mass by running a linear model with gut mass as the dependent variable, fiber and stress factors and their interaction as the independent variables, and body mass as the covariate. We then explored the possible effect of the experimental factors on the physiology of larvae by running linear models with relative corticosterone concentration (i.e., corrected for body mass), oxidative stress parameters (calatase, glutathione reductase, superoxide dismutase, glutathione peroxidase, lipid peroxidation) or relative telomere length as dependent variables, and fiber and stress factors and their interaction as independent variables. When we detected a significant effect of either a single factor or the interaction between both factors, we conducted post hoc Tukey tests to identify differences between levels of the different factors using the function *emmeans* (package *emmeans*). To meet parametric assumptions, we log‐transformed gut mass, relative corticosterone concentration, levels of oxidative stress parameters, and relative telomere length.

## 3 Results

**Figure 1:**
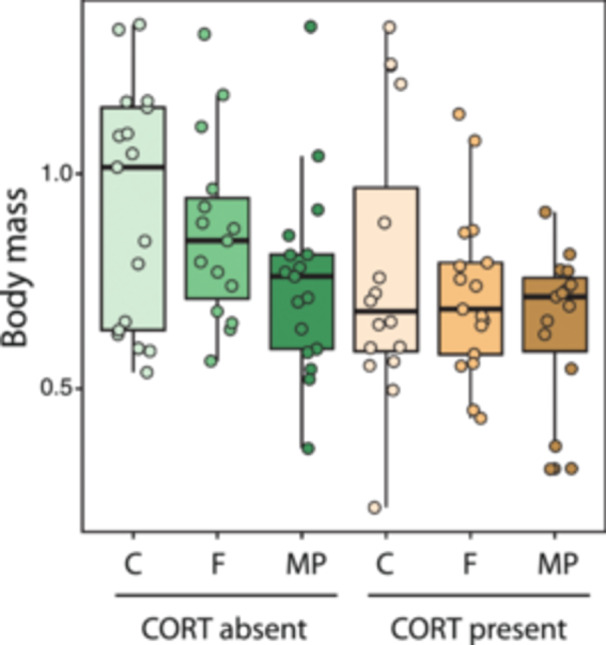
Variation in body mass in *Xenopus laevis* larvae exposed either to the presence or absence of exogenous corticosterone (100 nM CORT), combined with three different levels of fiber exposure (control with no fibers –C–, cellulose fiber –F–, or microplastic –MP– treatments, see Section 2 for details). Boxes represent 25th–75th percentiles, lines within boxes represent median values, and vertical lines represent maximum and minimum data values. Larval mass was significantly reduced by fiber type and stress exposure, with no significant interaction. Microplastics reduced larval mass compared to controls by 17.98%, while cellulose had no effect. CORT exposure reduced larval mass by 15.49%.

Fiber (*F*_2,91_ = 3.34, *p* = 0.040) and stress exposure (*F*_1,91_ = 7.03, *p* = 0.009) induced a reduction in larval body mass (Figure 1), whereas their interaction was not significant (*F*_2,91_ = 0.07, *p* = 0.931; Figure 1). MPs reduced larval body mass by 17.98% on average, as compared to control conditions (Tukey test = 0.034; Figure 1), whereas cellulose fiber did not affect larval body mass (Tukey test = 0.273; Figure 1). Likewise, CORT exposure reduced larval body mass by 15.49%, on average (Figure 1). SVL was also reduced by CORT (*F*_1,96_ = 7.76, *p* = 0.006), whereas fiber exposure or the interaction between both factors did not affect SVL (both *p* > 0.084). Gut mass, controlled for overall body mass, was reduced in response to CORT exposure by 25.33% on average (*F*_2,90_ = 11.01, *p* = 0.001), and it was unaffected by fiber exposure or the interaction between both factors (both *p* > 0.245).

**Figure 2:**
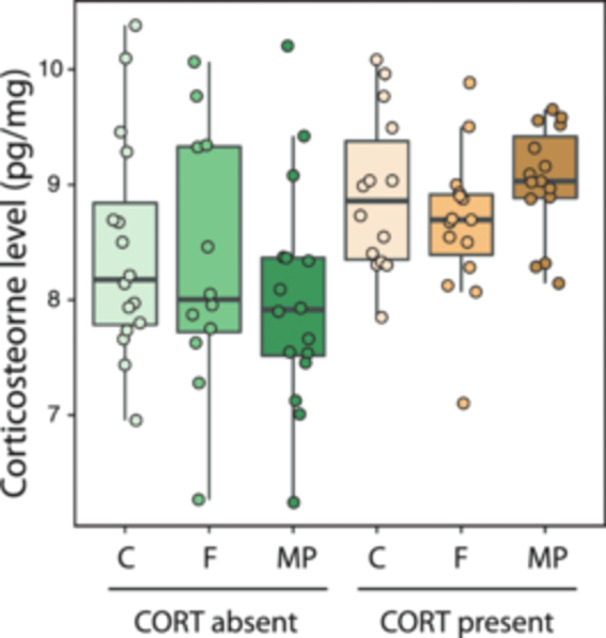
Variation in corticosterone concentration measured in tail tissue of *Xenopus laevis* larvae exposed either to the absent or present of corticosterone (CORT) exogenously added to the water (100 nM), combined with three different levels of fiber exposure (control with no fibers –C–, and cellulose fiber –F–, and microplastic –MP– treatments, see Methods for details). Boxes represent 25th–75th percentiles, lines within boxes represent median values, and vertical lines represent maximum and minimum data values. Larvae exposed to exogenous CORT had a significantly higher level of CORT in the tail tissue by 7.40%. Fiber type and the interaction between fiber and stress exposure had no effect on CORT levels.

**Figure 3:**
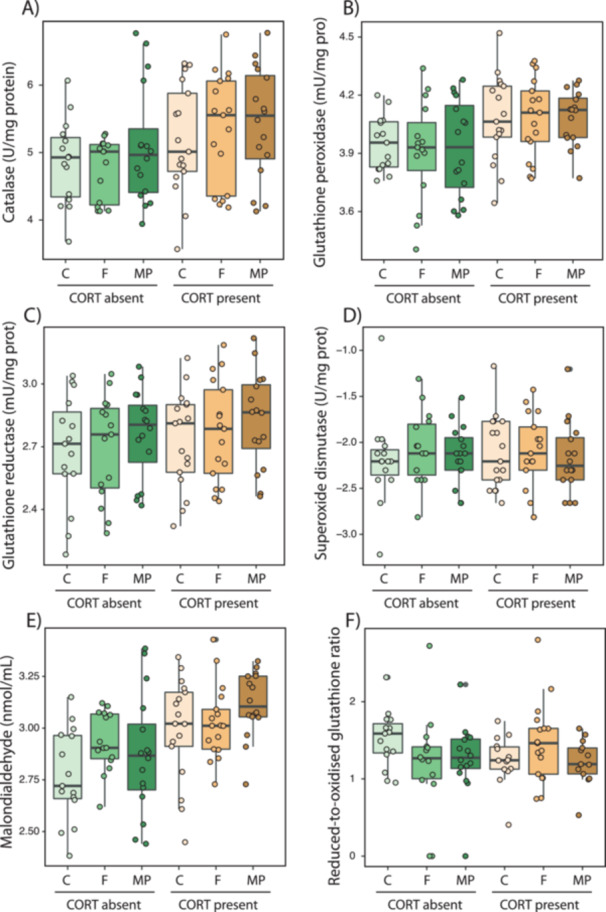
Variation in oxidative stress parameters. Antioxidant enzymes: (A) catalase, (B) glutathione peroxidase, (C) glutathione reductase, and (D) superoxide dismutase. Oxidative damage in lipids: (E) malondialdehyde content. Nonenzymatic antioxidant status: (F) reduced‐to‐oxidized glutathione). All these paremeters were measured in the liver of *Xenopus laevis* larvae exposed either to the absent or present of corticosterone (CORT) exogenously added to the water (100 nM), combined with three different levels of fiber exposure (control with no fibers –C–, and cellulose fiber –F–, and microplastic –MP– treatments, see Methods for details). Boxes represent 25th–75th percentiles, lines within boxes represent median values, and vertical lines represent maximum and minimum data values. Exogenous CORT significantly increased catalase activity, glutathione peroxidase activity, and lipid peroxidation levels. Fiber type and its interaction with stress had no effect on these markers.

**Figure 4:**
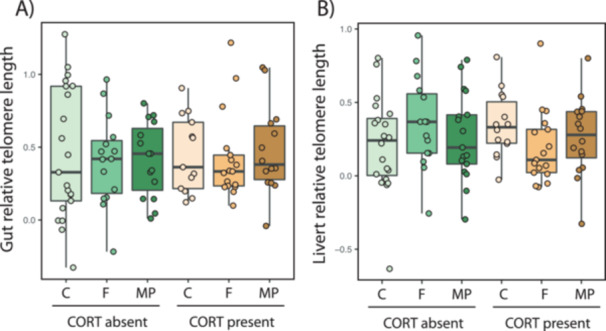
Variation in relative telomere length in the (A) gut and (B) liver of *Xenopus laevis* larvae exposed either to the absent or present of corticosterone (CORT) exogenously added to the water (100 nM), combined with three different levels of fiber exposure (control with no fibers –C–, and cellulose fiber –F–, and microplastic –MP– treatments, see Methods for details). Boxes represent 25th–75th percentiles, lines within boxes represent median values, and vertical lines represent maximum and minimum data values. Telomere length was not affected by any of the experimental factors.

Expectedly, larvae exposed to exogenous CORT had higher levels of the hormone in tail tissue (by 7.40% on average; *F*_1,82_ = 12.40, *p* < 0.001; Figure 2), but fiber exposure or the interaction between fiber and stress factors did not result in altered CORT levels (*F*_2,90_ = 0.52, *p* = 0.598 and *F*_2,90_ = 1.33, *p* = 0.268, respectively; Figure 2). Likewise, exogenous CORT increased the activity of the antioxidant enzymes catalase (*F*_2,90_ = 7.30, *p* = 0.008; Figure 3) and glutathione peroxidase (*F*_2,90_ = 12.37, *p* < 0.001; Figure 3), and the levels of lipid peroxidation (*F*_2,90_ = 18.07, *p* < 0.001; Figure 3). Fiber exposure or the interaction with exogenous CORT did not incur in redox alterations regarding the activities of antioxidant enzymes (all *p* > 0.737) or the markers of oxidative damage or status (malondialdehyde levels and the reduced‐to‐oxidized glutathione ratio, respectively; all *p* > 0.093). Finally, none of the experimental factors or their interaction caused significant reductions in telomere length, in either the liver or the gut (Figure 4; all *p* values > 0.334).

## 4 Discussion

Microplastics (MPs) are expected to cause detrimental effects on animal development and health, however, the severity of the effect will vary across taxa and MPs load, and the mechanisms by which MPs can be harmful are not yet fully understood. Here we found evidence that MP exposure substantially reduced growth rate of the filter‐feeding larvae of *X. laevis*. However, despite their impact on growth rate, we did not observe major changes in physiological parameters associated with stress status (i.e., corticosterone levels), or health and ageing (i.e., redox balance and telomere length). Our findings, therefore, suggest that environmentally realistic concentration of MPs may have small impact on stress physiology even though they result in considerable growth related carry‐over effects in amphibian larvae (but see: Ruthsatz, Schwarz, et al. 2023).

Coping with pollutants is often energetically demanding and can disrupt homeostasis in organisms inhabiting terrestrial or aquatic habitats (Noyes et al. 2009; rev. in Rohr et al. 2011). While eco‐toxicological research has identified the causes and consequences of being exposed to some pollutants (García‐Fernández et al. 2020; Michelangeli et al. 2022; Saaristo et al. 2018), further exploration is still needed in many other cases such as MP pollution. MPs were first identified in sea water in the 1960s (Bergmann et al. 2015; Carpenter and Smith 1972) and, from that moment onwards, plastic litter has been found across marine, terrestrial, and atmospheric environments (Jiang 2021; Prokić et al. 2019). Although MP exposure is expected to impair animal growth, development, or fecundity, evidence is equivocal across taxa (Chang et al. 2022; Prata et al. 2021; Zolotova et al. 2022). Our study shows a negative impact of MP exposure on the body mass of the filter‐feeding larvae of *X. laevis*, in accordance with other studies on aquatic organisms (rev. in Burgos‐Aceves et al. 2022). Similar to other ectotherms, body mass is considered a good proxy for survival in amphibians, particularly at metamorphic stages, with smaller individuals often experiencing lower survival odds later in life (Berven 1990; Cabrera‐Guzmán et al. 2013; Gomez‐Mestre and Tejedo 2003; Smith‐Gill and Berven 1979). Intriguingly, we observed a lack of variation in skeletal growth (i.e., body length) in larvae exposed to MPs. It is plausible that higher (but unrealistic) MP concentrations are needed to cause reductions in skeletal growth, and the interplay between MPs and other conditions such as temperature may also play a role here (Carreira et al. 2016). Additionally, it is possible that developmental plasticity in intestinal morphology such as increased gut length might have allowed for a compensation in skeletal growth as this is a mechanism often found in response to low food quality intake across taxa including amphibians (Ruthsatz, Giertz, et al. 2019). If MPs are ingested together with the actual food source, the protein and energy density of the diet is reduced as MP particles are truly non‐digestible fibers and lack any nutrients or energy that could be assimilated. In a previous study, we demonstrated that developmental plasticity resulting a longer intestinal tract allowed for such a growth compensation in body mass but not body length when *Xenopus* larvae were exposed to a lower food quality through the ingestion of cellulose or MP fibers (Ruthsatz et al. 2022). However, MP exposure did not alter the mass of larval gut in the present study, suggesting that the response to MPs in terms of gut plasticity could be genotype or context dependent.

Once confirming the negative effects of MPs on the growth of *X. laevis* larvae, we explored whether this process impaired their stress physiology. Chronically stressed animals can either upregulate or downregulate glucocorticoid levels (Rich and Romero 2005; Romero and Beattie 2022; Wingfield 2013). In our study, larvae exposed to MPs for 2 weeks, did not experience changes in their CORT levels. This result could indicate that the MP concentration used in our study might not have been enough to induce metabolic alterations and to activate the hypothalamic‐pituitary‐interrenal axis and the subsequent glucocorticoid release in *Xenopus* larvae. Alternatively, larvae could have experienced unbalanced CORT levels soon after the exposure to MPs, but managed to recover basal hormone levels shortly afterward. However, since tadpoles were measured at only a single sampling point in this study, subtle changes in CORT levels may not have been detected. Tracking glucocorticoid dynamics in animals coping with MPs (and other pollutants) will help understand the actual impact of this relatively novel contaminant on the stress status of wildlife.

As expected, we observed that simulated stress through exogenously added CORT resulted in oxidative damage and elevated antioxidant responses in *Xenopus* larvae. In contrast, plastic pollution did not induce such changes in the redox status of larvae, not even in an interactive manner with previously physiologically stressed individuals. Based on meta‐analytical evidence, pollution is known to lead to antioxidant responses or oxidative damage in several taxa, including amphibians (Chatelain et al. 2020; Isaksson 2010; Martin et al. 2024). This also seems to be the case for MPs, however, oxidative stress changes may only occur in the short term or be dependent on MP concentration (Li et al. 2022; Li et al. 2023). Unlike the redox status, telomere dynamics have been overlooked in MP studies, which is surprising regarding the vast use of this ageing marker in eco‐toxicological research (Chatelain et al. 2020; Salmón and Burraco 2022). In amphibians, knowledge of telomere length variation in organisms coping with pollutants is, so far, restricted to a few studies (e.g., Cheron et al. 2022; Sabol et al. 2024; Zamora‐Camacho et al. 2023), often observing little to none changes in telomere length. Likewise, in our experiment, MP exposure did not result in shortened telomeres, also in accordance with the observed lack of variation in CORT levels or oxidative stress. This result suggests that environmentally relevant concentration of MPs do not alter accelerate ageing rates in *X. laevis* larvae. Alternatively, reductions in larval growth might have resulted in reduced cell division and less demanding metabolic processes required for somatic maintenance, which could have finally resulted in unaltered telomeres. This possibility should be explored through estimates of cell division rates and aerobic (mitochondrial) metabolism across tissues.

## 5 Conclusions

Our study confirms that the exposure to an environmentally realistic concentration of MPs reduces growth in filter‐feeding amphibian larvae. Such growth effects do not include consequences for stress and ageing‐related physiological mechanisms, suggesting minor carry‐over effects of MP exposure. Overall, this study improves our mechanistic understanding of the effect of MPs on the life history and physiology of aquatic organisms, a knowledge that could be integrated into mechanistic niche modeling to finally develop effective conservation actions (e.g., Burraco, Lucas, et al. 2022; Kearney and Porter 2009; Newman et al. 2022).

## Author Contributions

**Colette Martin:** data curation (supporting), methodology (equal), investigation (equal), writing – original draft (equal), writing – review and editing (equal). **Katharina Ruthsatz:** conceptualization (lead), supervision (equal), methodology (equal), data curation (equal), formal analysis (supporting), investigation (equal), writing – original draft (equal), writing – review and editing (equal), project administration (equal), funding acquisition (lead). **Ivan Gomez‐Mestre:** conceptualization (equal), resources (supporting), writing – review and editing (equal), funding acquisition (supporting). **Pablo Burraco:** conceptualization (equal), supervision (equal), formal analysis (lead), investigation (equal), methodology (equal), project administration (equal), writing – original draft (equal), writing – review and editing (equal), funding acquisition (supporting).

## Ethics Statement

All experimental procedures were conducted at Doñana Biological Station (Spanish National Research Council, CSIC, Seville, Spain) following the approved bioethical permit 12/12/2023/108.

## Conflicts of Interest

The authors declare that the research was conducted in the absence of any commercial or financial relationships that could be construed as a potential conflicts of interest.

## Data Availability

The data set of this study can be accessed at https://doi.org/10.6084/m9.figshare.25958923.v1.
